# Evaluating the Feasibility and Cost-Effectiveness of EzeCheck for Hemoglobin Screening Across India

**DOI:** 10.7759/cureus.72945

**Published:** 2024-11-03

**Authors:** Harshavardhan Rajagopal, Nirmal K Mohakud, Dayanidhi Meher, Balu PS, Sonal Deep Sharma

**Affiliations:** 1 General Surgery, American Hospital Dubai, Dubai, ARE; 2 Pediatric Medicine, Kalinga Institute of Medical Sciences, Bhubaneswar, IND; 3 Endocrinology, Kalinga Institute of Medical Sciences, Bhubaneswar, IND; 4 Community Medicine, Subbaiah Institute of Medical Sciences, Shimoga, IND; 5 Public Health, SARU Foundation, Noida, IND

**Keywords:** ezecheck, ezerx, feasibility, hemoglobin: hb, hemoglobinometer, non-invasive

## Abstract

Background

Anemia, particularly due to low hemoglobin levels, is a significant public health concern in India. Traditional invasive screening methods can be burdensome and may limit access to diagnosis. This study evaluated the feasibility and cost-effectiveness of a noninvasive hemoglobin screening device called EzeCheck for determining hemoglobin levels across diverse populations in India. We aimed to assess the user feasibility of the EzeCheck device and to conduct a comparative analysis of its cost-effectiveness against other available invasive and noninvasive methods to determine hemoglobin levels.

Methods

This study adopted a mixed-methods approach, incorporating both quantitative and qualitative data for analysis. User feedback was collected through surveys and interviews with healthcare professionals and workers across various regions. A cost-effectiveness analysis compared the EzeCheck device with other available noninvasive devices and standard invasive methods, accounting for factors such as equipment costs, consumables requirements, time efficiency, and overall testing costs.

Results

Preliminary findings suggest that the EzeCheck device was well-received by users, with 198 (86%) healthcare professionals reporting improved workflow and patient satisfaction. The comparative analysis revealed that the EzeCheck device was the most affordable option in terms of overall screening costs, while also increasing screening efficiency to a great extent. Moreover, it provided a safer and more comfortable experience for patients and users.

Conclusions

The noninvasive EzeCheck hemoglobin screening device demonstrates superior feasibility and cost-effectiveness over traditional methods. This study highlights its potential for enhancing anemia detection in India, particularly in underserved areas. By improving accessibility and reducing costs, this device could significantly impact public health outcomes and inform future health policy initiatives.

## Introduction

Hemoglobin measurement is a critical component of diagnosing and managing various hematological conditions, including anemia, a widespread global health issue affecting millions of individuals, particularly in resource-limited settings. Early and accurate detection of anemia, a condition characterized by deficiency in the quantity or quality of red blood cells, is crucial for effective patient management and improved health outcomes [1,2]. Traditional methods for assessing hemoglobin levels typically involve invasive techniques such as venipuncture or fingerstick blood tests, which can be uncomfortable and logistically challenging, particularly in remote or resource-limited settings [3].

In recent years, there has been growing interest in the development of noninvasive technologies with the potential to streamline and enhance the accessibility of hemoglobin screening. Among these innovations, certain devices such as digital hemoglobinometers (e.g., TrueHb and HemoCue) and noninvasive technologies such as Masimo Pulse Oximetry, Orsense NBM 200, and other spectroscopy-based devices have shown promise for public health applications [4]. Evaluating these devices is essential for identifying the most cost-effective and user-friendly options and ensuring that these technologies contribute to a sustainable healthcare future.

Feasibility studies play a vital role in determining whether the abovementioned devices can be effectively integrated into anemia screening programs and provide tangible benefits [5]. The EzeCheck device represents a promising advancement in this field. The feasibility of integrating noninvasive hemoglobin screening into routine clinical practice hinges on several critical factors, including the accuracy of the device, its ease of use, pocket-friendliness, and its ability to function effectively across diverse populations and environmental conditions.

Cost-effectiveness is another critical dimension of device evaluation. The cost of acquiring, operating, and maintaining diagnostic equipment plays a significant role in treatment decision-making for healthcare facilities. For EzeCheck, this process involves analyzing the costs of the device associated with any necessary training or support. Furthermore, effective hemoglobin screening and timely intervention can reduce the incidence of severe anemia and its complications, potentially lowering overall healthcare costs and improving patient outcomes.

EzeCheck

Developed by EzeRx Health Tech Pvt. Ltd. (Bhubaneswar, India), EzeCheck is a noninvasive hemoglobin screening device that is designed to provide accurate and rapid hemoglobin assessments without the need for blood samples [6]. EzeRx is a med-tech start-up company that develops innovative and affordable healthcare solutions by incorporating artificial intelligence, machine learning algorithms, and Internet of Things integration, aiming to revolutionize preventive healthcare and foster early detection. The EzeCheck device utilizes absorption spectroscopy to measure hemoglobin levels by penetrating a beam of light through the dermal layer of the fingertip. EzeCheck is operated by a mobile-based Android app, which carries out the functionalities of the device, beginning from the entry of the patient’s details to the display of the hemoglobin values. The app is available in English as well as vernacular languages (e.g., Hindi, Odia, and Assamese) for user convenience. The app also features a record section, which enables the user to fetch their old reports and store them in a single place. Being noninvasive, EzeCheck is especially useful for determining hemoglobin levels in children.

Our study investigates the reliability and cost-effectiveness of EzeCheck compared to other invasive and noninvasive hemoglobin measurement methods, as the accuracy of the device has already been reported in a different publication [7]. Similarly, the impact of regular hemoglobin testing using the EzeCheck device for addressing anemia is well known [8]. Additionally, we assess user satisfaction, device usability, and the practical challenges of implementing this technology in various healthcare settings. By addressing these key elements, this research seeks to determine the potential of the EzeCheck device to transform hemoglobin screening practices and improve health outcomes through more accessible and less invasive testing options. Through this work, we aim to contribute to the growing body of evidence supporting innovative solutions to global health challenges, which will be valuable information for healthcare providers, policymakers, and public health organizations toward anemia eradication programs.

## Materials and methods

In this study, we assessed the feasibility and cost-effectiveness of EzeCheck compared to other devices. With this comparative study, we aimed to determine the maximal benefits of all the devices and enhance their impact on the development of healthcare amenities in remote areas. The samples chosen were selected from the customer database of EzeCheck users and from other field users of the device.

For determining the cost-effectiveness of EzeCheck, different technology-based hemoglobin testing devices were reviewed, and telephonic interviews of various EzeCheck users across both private and government organizations were conducted to conduct a feasibility study. These interviews were gathered to provide insights into EzeCheck’s user-friendliness, usefulness, and suitability for remote settings.

The cost-effectiveness analysis was performed utilizing both invasive and noninvasive methods for anemia detection using established protocols. Current evidence suggests that digital hemoglobinometers such as HemoCue and TrueHb offer superior performance in anemia detection, with HemoCue being particularly cost-effective for general anemia detection and TrueHb for severe anemia. Future advancements in technology will require ongoing health technology assessments for device re-evaluation [9].

Feasibility study

**Figure 1:**
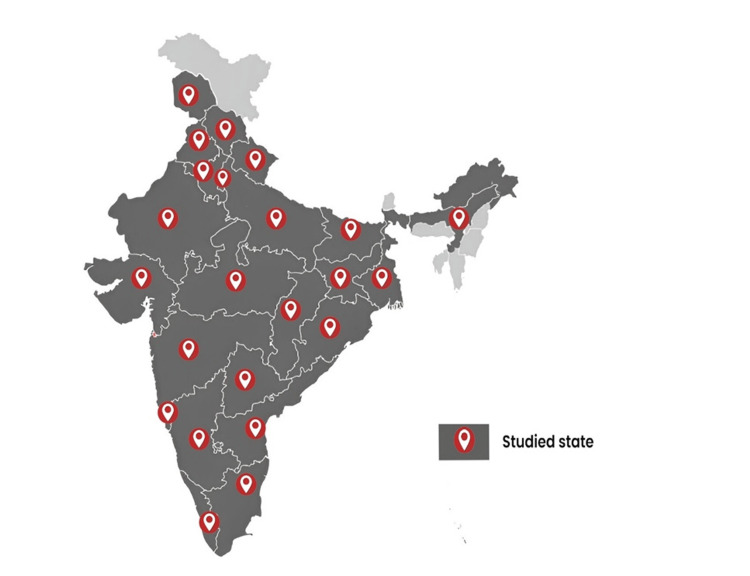
Representation of the states from which the study participants were drawn

A cohort of 230 individuals was taken across India from the EzeRx customer database. From each state (23 states were included), 10 users were randomly selected for telephonic conversations. The states covered in this study are represented in Figure 1. Participants were excluded if they refused to participate in the study. The term “feasibility” in this study describes how feasible it is to use the device for determining hemoglobin levels in a certain healthcare setting. Participants with varied educational qualifications and professions were included in this study to determine the ease of use in device operation with respect to the user’s educational qualification.

Cost-effectiveness

A comparative study was conducted by studying different aspects of the hemoglobin devices that could affect the usability and cost expenditure of the testing process. Factors such as ease in conducting the test, method (invasive or noninvasive), use of consumables, and recharge requirements were considered for the cost comparison, along with the overall price of the equipment/device. Pricing details were obtained from different official websites of the hemoglobin device manufacturers and via online shopping portals (e.g., IndiaMART) for all hemoglobin devices and equipment that are available in the Indian market. Research data were captured and analyzed to establish a comparison between the available devices. In this study, cost-effectiveness involved evaluating the economic viability and efficiency of the studied device, which will assist in defining its usability, particularly for those residing in remote areas where affordability is a huge factor.

Each participant was well aware of the testing procedure for hemoglobin using the EzeCheck device, as they received prior training sessions from appropriate representatives of EzeRx. Participants were asked to rate the device from 1 to 10 (1 = extremely poor and 10 = extremely good) based on the prepared questionnaire and were also asked to provide any pertinent comments/remarks about the ease of using the device from their individual perspective. Participants were also asked whether they were completely aware of all the functionalities of the app and comfortable enough to use the app in their respective native languages.

Data collection

In addition to the perception-based questionnaire, baseline demographic details were obtained for each participant, including name, gender, highest educational qualification, and their respective designation. An interview-based approach was implemented for eliciting the quantitative data orally from the participants by means of telephonic conversation, where the participants were asked (in their local language) about EzeCheck’s feasibility based on their experience with using the device. By utilizing the unified theory of acceptance and use of technology (UTAUT) [10], we developed a survey-based questionnaire for determining each participant’s acceptance and view of the feasibility of digitalized healthcare technologies like EzeCheck. The questionnaire used a Likert scale to score the user’s acceptance of the EzeCheck device for determining hemoglobin levels with a noninvasive approach.

Authors BPS and SDS were provided with the questionnaire, which was prepared after a collective discussion among all the authors. Both the authors conducted the interviews in a private space, and each participant was interviewed for five to 10 minutes. For data analysis, the interview data were entered into a spreadsheet in order to perform statistical calculations through Microsoft Excel 2021 (Microsoft Corporation, Redmond, WA, USA). For determining cost-effectiveness, the collected data from online sources were placed in tabular form for easy and effective comparison.

Ethical considerations

We obtained ethical clearance to perform this study from the Institutional Ethical Committee of Kalinga Institute of Medical Sciences, Bhubaneswar, India (approval number KIIT/KIMS/IEC/543/2021). We implemented all necessary measures to protect the privacy and confidentiality of the participant’s data.

## Results

A total of 230 participants (10 individuals each from 23 different states across India) participated in this study. All participants reported that the EzeCheck device was more user-friendly compared to conventional lab-based hemoglobin tests, highlighting its speed, ease of use, and pain-free nature.

Of the 230 participants, 172 (74.78%) were male, and 58 (25.22%) were female. The highest educational level among the participants was postgraduate level, accounting for 48 (20.87%) participants, whereas only two (0.87%) participants listed primary school as their educational level. Most of the participants, 143 (62.17%), listed graduation as their highest level of education. The demographic characteristics of the participants are represented in Table 1.

**Table 1:** Demographic characteristics of the participants (N = 230) ANM: auxiliary nurse midwifery; CHO: community health officer

Characteristic		n (%)
Gender	Male	172 (74.78)
	Female	58 (25.22)
Educational level	Primary school	02 (0.87)
	Higher secondary	10 (4.35)
	Diploma	27 (11.74)
	Graduation	143 (62.17)
	Post-graduation	48 (20.87)
Designation	ANM/field worker/territory manager	74 (32.17)
	Block coordinator/CHO/field supervisor	17 (7.40)
	Regional manager/area business manager	134 (58.26)
	Other (e.g., director and doctor)	5 (2.17)

Regarding user satisfaction, 167 (72.61%) participants rated EzeCheck between 8 and 10, indicating high satisfaction with its effectiveness and ease of use for hemoglobin screening; 50 (21.74%) participants rated the device between 6 and 7.9, reflecting moderate satisfaction; and 13 (5.65%) were less satisfied, giving ratings below 6.

When asked about the ease of the EzeCheck testing process as compared to invasive methods, 208 (90.44%) participants expressed high satisfaction with the convenience of the device, 17 (7.39%) rated it as moderately convenient (between 6 and 7.9), and only five (2.17%) found it inconvenient (rated below 6). Regarding societal acceptance and individuals’ willingness to test with EzeCheck, 159 (69.13%) participants reported that users were likely to participate (rated 8 and above) due to the test’s noninvasive nature. In contrast, 46 (20%) indicated that only a moderate (rated between 6 and 7.9) number of individuals were willing to test with EzeCheck, and 25 (10.87%) participants noted a general lack of interest (rated below 6) among individuals for the noninvasive testing method. In terms of the time to complete the test with the app and displaying the results, 142 (61.74%) participants rated this process as 8 and above, 69 (30%) rated between 6 and 7.9, and 19 (8.26%) dissatisfied with the app completion time and rated below 6. Details of the responses to the other interview questions are provided in Table 2. The participants’ concerns and suggested areas of improvement are presented in Table 3.

**Table 2:** Participant questionnaire responses on the feasibility of EzeCheck usage (N = 230)

Questionnaire item	n (%)		
	Rated <6	Rated 6-7.9	Rated ≥8
Ease of testing process as compared to the invasive method	5 (2.17)	17 (7.4)	208 (90.43)
Pregnant women are willing to take the noninvasive EzeCheck test under Pradhan Mantri Matru Vandana Yojana (PMMVY) [11]	4 (1.74)	14 (6.09)	212 (92.17)
Children are willing to take the noninvasive EzeCheck test under the School Health Programme [12,13]	5 (2.17)	27 (11.74)	198 (86.09)
Percentage of society that accepts this technology and is willing to take the test	25 (10.87)	46 (20)	159 (69.13)
Ease in connecting the device with the mobile-based app	21 (9.13)	36 (15.65)	173 (75.22)
Ease in switching ON/OFF the device	3 (1.30)	14 (6.09)	213 (92.61)
Ease in finger placement on the device and the process of conducting the test	19 (8.26)	36 (15.65)	175 (76.09)
Ease in accessing and understanding the PDF report generated after test completion	6 (2.61)	17 (7.39)	207 (90)
Ease in charging the device after use	4 (1.74)	16 (6.96)	210 (91.30)
Ease in understanding the error codes and retaking the test	14 (6.09)	65 (28.26)	151 (65.65)
Ease in connecting with the company’s customer support and getting a timely resolution to concerns	15 (6.52)	34 (14.78)	181 (78.70)
Completion time of the test	19 (8.26)	69 (30)	142 (61.74)
Will you recommend others to undergo noninvasive tests using EzeCheck?	14 (6.09)	54 (23.48)	162 (70.43)

**Table 3:** Participants’ concerns and recommendations for EzeCheck (N = 104) Generic responses are listed for all concerns/recommendations.

Category	Concern/recommendation	n (%)
Bluetooth connection	Sometimes, due to inappropriate Bluetooth connectivity, the device fails to respond, so we need to restart the device and try connecting it with the app.	15 (14.42)
Test completion time	The app takes more than a minute to complete some of the tests.	8 (7.69)
Overall functionality	Very convenient to use and can be easily carried to any location.	45 (43.27)
Overall functionality	The device is unique, and management must continue to provide usage of the device.	11 (10.58)
Finger placement	Error codes are displayed for some patients, so care is necessary while placing the finger on the device.	21 (20.19)
App language	Other regional languages must be included in the app for easy understanding.	4 (3.85)

## Discussion

The potential of noninvasive medical devices to enhance patient comfort, streamline procedures, and reduce overall healthcare costs underscores their viability and cost-effectiveness in modern healthcare environments. Noninvasive devices are more feasible and cost-effective than invasive devices for many reasons: they are pain-free, eliminate medical waste, require minimal or no setup, and provide rapid results, which can facilitate early disease detection.

Estimating hemoglobin levels through invasive methods presents significant challenges, particularly for individuals residing in remote areas with limited healthcare access. This approach is hindered by several drawbacks. For example, some of these devices (e.g., hematology analyzer) are often non-portable or require proper medical setup to operate, the results are not immediately accessible (three to four hours are required for sample processing), and the overall process incurs substantial costs (e.g., reagent cost, technician salary, and machine maintenance) [14,15]. These bottlenecks underscore the critical need for noninvasive methods for hemoglobin estimation to address these challenges effectively. With advancements in healthcare and AI technologies, numerous commercially available, noninvasive hemoglobin measurement devices have been developed. However, such devices are not readily available in Indian markets. These noninvasive devices comprise various technologies, from simple algorithmic calculations to large, AI-based spectroscopic methods. Although various body parts (e.g., fingertip, nail bed, and lower eyelid) are used for measuring hemoglobin values using advanced signal processing techniques and machine learning algorithms, a clinically acceptable accuracy range was observed with devices involving the use of the fingertip [16-18]. A comparative study between invasive (HemoCue) and noninvasive (Orsense NBM 200) hemoglobin estimating devices demonstrated that noninvasive technology can significantly reduce the need for finger pricking and eliminate biomedical waste, making it a valuable tool for hemoglobin estimation [19]. Similarly, another study involving the use of the Pronto-7 pulse CO-oximetry device (Masimo Corporation) for determining hemoglobin levels in preoperative assessment highlighted the significance of noninvasive technologies, as they enable faster anemia diagnosis and management at the time of the initial clinical visit [20].

In the field of noninvasive devices, EzeCheck offers superior portability with wireless technology (being Bluetooth enabled), an interactive mobile dashboard with clinical statistics representing anemia count (i.e., the number of anemic cases observed based on the overall conducted tests using the EzeCheck device), and easy retrieval and traceability of reports, even years after testing, making it a suitable option for this study. To our knowledge, this is the first study to evaluate the feasibility and cost-effectiveness of EzeCheck, which will assist in utilizing such devices for effective and affordable hemoglobin screening at large scales.

Our findings indicate that the EzeCheck device is highly feasible for both operators and patients due to its noninvasive nature and prompt reporting capabilities. Community acceptance was notably high, with 69.13% of participants expressing positive feedback. Specifically, 86% of participants reported that children were receptive to the test, and 92% reported acceptance of the EzeCheck device by pregnant women for regular hemoglobin screening purposes. Participants reported that the EzeCheck device enables rapid and efficient screening of large populations, as compared to traditional invasive hemoglobin testing methods. Moreover, the device’s app, featuring a record section, simplifies record-keeping of the conducted tests, reducing manual effort and saving time. All participants demonstrated familiarity with the app’s functionalities and found them valuable for device operation. The most common concerns of the participants were delays in test completion, societal acceptance, and proper finger placement. As a noninvasive method for hemoglobin estimation, this technology is susceptible to various factors that can affect its performance, such as suboptimal finger placement, compromised blood circulation, and low body temperature. These factors may impact the accuracy and convenience of the device for some users. Nevertheless, the adoption of these noninvasive and portable devices has significantly reduced the requirement of patients to travel to urban hospitals for hemoglobin testing, saving time and transportation costs [21].

Regarding cost-effectiveness, we found that the EzeCheck device is economical, with a per-test cost of less than 10 INR, making it more affordable than other testing methods. Additionally, its noninvasive nature eliminates the need for consumables and avoids the generation of biomedical waste. Although the Masimo Rad-67 Spot Check device offers similar benefits, its cost is approximately five times higher than that of the EzeCheck device, rendering it less suitable for resource-constrained settings [22]. Various hemoglobin estimation methods commonly available in India are compared in Table 4. Although spot-check devices such as HemoCue are considered the second-most cost-effective option for hemoglobin testing due to their rapid results, they require a significant initial investment and incur ongoing operational expenses. Furthermore, the consumables for these devices have a relatively short shelf life of approximately two years [23]. Similarly, although Sahli’s hemoglobinometer is cost-effective, it shows notable variability in diagnostic accuracy due to differences in the capillary blood sampling procedure [24-26], which restricts its utility in major hospitals and clinics; furthermore, the need for a blood sample, even if obtained through a simple fingerprick, restricts its use primarily to healthcare settings.

**Table 4:** Cost comparison between different invasive and noninvasive methods of hemoglobin estimation Hb: hemoglobin; INR: Indian rupees

Feature	EzeCheck	Sahli’s method	HemoCue	Hematology analyzer	TrueHb	Masimo Rad-67 Spot Check
Method of testing	Noninvasive	Invasive	Invasive	Invasive	Invasive	Noninvasive
Required consumables	None	Comparator holder, sample tube, and pipette	Microcuvettes	Reagents, pipettes, and sample tubes	Test strips	None
Generates biomedical waste	No	Yes	Yes	Yes	Yes	No
Required time for report generation	<1 minute	20-30 minutes	<1 minute	4-5 hours	<1 minute	<1 minute
Device/equipment cost (in INR)	35,000-40,000	1,200-1,500	80,000-90,000	≥600,000	5,000	90,000-100,000
Cost per Hb test (in INR)	<10	<10	>27	>50	>15	>50
Additional costs	Not applicable	Applicable	Applicable	Applicable	Applicable	Not applicable
Requires management of biomedical waste	No	Yes	Yes	Yes	Yes	No
Disease burden cost due to fear factor	Not applicable	Applicable	Applicable	Applicable	Applicable	Not applicable

Invasive testing methods, which rely on blood sample collection and the use of various reagents, cotton, syringes, and other materials, generate significant amounts of medical waste. This waste requires proper disposal and management, contributing to additional costs and labor. Furthermore, the discomfort associated with invasive procedures often deters large segments of the population, particularly children, from undergoing testing. This reluctance can lead to delays in diagnosis and progression of the disease, resulting in increased severity and a substantial financial burden on families once the condition is finally diagnosed. In contrast, noninvasive devices eliminate the need for waste management and reduce the financial impact on families, offering a significant advantage in addressing these concerns.

For a device to be considered optimal for community-based programs, it must demonstrate validity in terms of accuracy, rapid operation, ease of use, user and patient friendliness, portability, reagent-free functionality, environmental sustainability, and broad operability. In this study, EzeCheck was found to be user-friendly, with its design and Bluetooth connectivity enhancing portability. Furthermore, its noninvasive nature eliminates the need for reagents or blood samples, thereby avoiding the generation of medical waste and positioning it as an effective, user- and environmentally friendly device for hemoglobin estimation.

Study limitations

The limitations highlighted in the study suggest a few critical areas for improvement in future research. Firstly, the restriction to a limited number of participants per state poses a challenge to the reliability and validity of the findings. A small sample size may not capture the full spectrum of experiences and perspectives within each state, leading to potential biases. Future research could benefit from larger sample sizes and more diverse participant selection to ensure that various demographics and socio-economic backgrounds are represented. This would enhance the robustness of the data and strengthen the overall conclusions.

Ultimately, a more extensive and inclusive study could not only provide richer insights into the topic but also inform policymakers and stakeholders more effectively, ensuring that interventions and programs are tailored to the diverse needs of the entire population in India.

## Conclusions

EzeCheck is a cost-efficient, noninvasive method for regular, large-scale screening of hemoglobin levels. From the user convenience perspective, the device is quite easy to use and provides valuable insights with its corresponding mobile app, which maintains historical data reports. Its compact and portable design, along with its Bluetooth connectivity, makes it an ideal option for use in resource-limited settings.
